# Comprehensive Transcriptomic Analysis of Brain Tissues From the Infarcted Area of MCAO Rats Revealed That Acupuncture Attenuates Brain Injury via the Complement System

**DOI:** 10.1002/brb3.70740

**Published:** 2025-08-21

**Authors:** Yanlin Liu, Yuting Jin, Yuxin Liu, Jiaqi Gao, Xiaomei Wang, Hongbin Ren, Yabo Hao, Xibin Yang, Kaitao Luo

**Affiliations:** ^1^ Acupuncture and Moxibustion Department Jiaxing Hospital of Traditional Chinese Medicine Affiliated With Zhejiang University of Traditional Chinese Medicine Jiaxing China; ^2^ Third Clinical Medical College Zhejiang Chinese Medical University Hangzhou China

**Keywords:** acupuncture, bioinformatics, inflammation, MCAO rats, stroke, transcriptomics

## Abstract

**Objective:**

This research focused on investigating acupuncture's effect on brain injury in middle cerebral artery occlusion (MCAO) rats and elucidating its potential mechanisms, with a focus on transcriptomic and protein‐level changes related to the complement system, which may be involved in acupuncture's therapeutic effects.

**Methods:**

An MCAO rat model was established and treated with acupuncture. Brain tissue from the infarct area was analyzed through RNA sequencing, Gene Ontology (GO), Kyoto Encyclopedia of Genes and Genomes (KEGG) enrichment, and protein–protein interaction (PPI) analyses, followed by qRT‐PCR and western blot validation of key complement‐related genes.

**Results:**

A total of 1792 differentially expressed genes (DEGs) were identified between the MCAO and acupuncture groups. Of these, 254 hub genes were associated with acupuncture's therapeutic effects. Seven complement‐related genes (C1QA, C1QB, C1QC, C2, C6, C7, and TREM2) were significantly downregulated by acupuncture. Functional enrichment revealed that these genes were involved in the inflammatory response, coagulation cascades, blood‒brain barrier regulation, and neutrophil extracellular trap (NET) formation.

**Conclusion:**

Our findings suggest that acupuncture alleviates stroke‐induced brain injury by modulating key components of the complement system and associated inflammatory pathways. These results provide mechanistic insights supporting the use of acupuncture as an adjuvant therapy for poststroke neuroinflammation.

## Graphical abstract

This study illustrates the mechanism by which acupuncture alleviates neurological deficits and reduces cerebral infarction in MCAO model rats. Transcriptomic and bioinformatics analyses revealed that acupuncture significantly downregulates key complement‐related genes (C1QA, C1QB, C1QC, C2, C6, C7, and TREM2), thereby inhibiting the classical complement pathway, inflammatory cytokine release, blood–brain barrier disruption, and oxidative stress. These molecular changes contribute to a smaller infarct area and improved neurological function. The findings suggest that acupuncture may exert neuroprotective effects through modulation of the complement system and related inflammatory pathways.

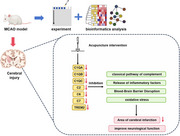

## 1 Introduction

As a disease that causes “thromboinflammation,” ischemic stroke (IS) has a high mortality and disability rates, posing a significant socioeconomic burden (Hong et al. 2022). After cerebral tissue ischemia‒reperfusion, there are large fluctuations in the oxygen and energy supply, which can cause severe damage to brain cells, and various pathophysiological processes, including cellular oedema, inflammation, blood‒brain barrier injury, and oxidative stress, are involved (Zhao et al. 2021). Most patients exhibit residual symptoms such as reduced limb movement, difficulty speaking, and cognitive impairment, which affect their quality of life (Hurford et al. 2020). At present, the main treatment methods for stroke are thrombolysis and neuroprotective drugs, which aim to save the dying neurons in the ischemic penumbra, but there is no treatment that effectively regulates neuroinflammation after stroke (Widimsky et al. 2023). Therefore, further study of neuroinflammation may provide novel ideas for adjuvant therapy for IS.

As a common clinical approach in China, acupuncture effectively treats multiple IS‐caused complications (Chavez et al. 2017; B. Li et al. 2022; L. Li, Xu, et al. 2023; N. Li, Wang, et al. 2023). Acupuncture not only enhances the recovery of limb movement recovery in stroke patients but also reduces the serum levels of inflammatory factors, inspiring our current research (Yanlin et al. 2024). The complement system functions as a fundamental effector of the immune response. The identification of intracellular complement has drawn growing scholarly attention to the physiological and pathological relationships between the central nervous system and the complement system (Kemper et al. 2023). Notably, the brain has been shown to be capable of synthesizing nearly all components of the complement system. The complement system participates not only in pathological processes such as stroke, neurodegeneration, cognitive impairment, and neuroinflammation but also in physiological processes including neuroprotection, synaptic pruning, and brain development (Bohlson and Tenner 2023). Clinical trials reveal that serum complement factor levels persist at elevated levels in IS patients for at least 7 days following disease onset (Ma et al. 2019). In middle cerebral artery occlusion (MCAO) mice, several complement‐related factors, such as C1q, C3a, and C5a, are also remarkably elevated in the brain tissues (Berkowitz et al. 2021). After ischemia, C1q is produced by local microglia. Inhibition of C1q can ameliorate blood‒brain barrier damage, reduce infarct size, and decrease the levels of inflammatory cytokines (Schartz and Tenner 2020). Therefore, regulating the complement system may be a strategy for reducing inflammation after stroke. However, whether the efficacy of acupuncture is related to the complement system remains to be explored.

Limited research has investigated how acupuncture influences brain gene expression and alleviates brain injury at the transcriptional level. This research investigated the transcriptomic changes in the ischemic area of MCAO rat brain tissue induced by acupuncture and performed bioinformatics analysis. Changes in the complement system after ischemia‒reperfusion were observed to explore whether the therapeutic effect of acupuncture on MCAO rats occurs through the complement system. This study reveals a potential mechanism through which acupuncture may exert therapeutic effects in IS, offering new insights and directions for IS treatment.)

## 2 Methods

### 2.1 Experimental Animals

Eighteen adult male Sprague‐Dawley (SD) rats (250–300 g) were provided and housed by Zhejiang Yingyang Pharmaceutical Research Co., Ltd., and all experiments were conducted in accordance with the approved experimental protocol. The animals were housed in a barrier housing facility under standard conditions (SPF grade), with three rats per cage. Under a 12‐h light–dark cycle, the rats were maintained in a temperature‐controlled setting (20 ± 2°C). Water and food were provided ad libitum. This study was approved by the Laboratory Animal Management and Welfare Ethical Review Committee of Zhejiang Yingyang Pharmaceutical Research Co., Ltd. All procedures adhered to the Guide for the Care and Use of Laboratory Animals of the National Institutes of Health. The MCAO model design was based on previously published studies (Chang et al. 2014).

### 2.2 Construction of the MCAO Model

Random grouping was conducted, dividing the rats into the MCAO, acu, and control groups, with six rats per group. An improved suture method was utilized for constructing the MCAO rat model (Y. Li and Zhang 2021). Briefly, rats underwent anesthesia through an intraperitoneal injection of Zoletil 50 (50 mg/kg). A heating pad, maintained at 37°C, was applied for stabilizing the rats' body temperature. Next, the rats were placed in a supine position to expose the right common carotid artery, external carotid artery, and internal carotid artery. A thread (0.26 ± 0.01 mm; A52636, Beijing Cinontech Co., Ltd., Beijing, China) was introduced through the common carotid artery into the internal carotid artery following external carotid artery ligation. The thread was left at a distance of 18–22 mm from the insertion point. The thread was removed after 90 min, and the wound was disinfected with iodine and closed. Ischemia‒reperfusion injury was evaluated after 24 h. Longa's 5‐point scale was utilized for scoring neurological deficits: 4 = *loss of spontaneous walking and consciousness*, 3 = *falling to the contralateral side*, 2 = *circling toward the contralateral side*, 1 = *inability to fully extend the affected forepaw*, and 0 = *no neurological impairment*. Only rats scoring 2 or 3 were enrolled in subsequent experiments.

### 2.3 Acupuncture Treatment

The rats in the acupuncture group were treated with disposable medical Huatuo brand stainless steel 0.18 mm × 10 mm acupuncture needles. Acupuncture was performed by an acupuncturist, following the National GB/T21709.4‐2008 Technical Operating Standard of Acupuncture, Part 4. The Xingnaokaiqiao acupoints Baihui (GV20), Shuigou (GV26), Neiguan (P6), and Sanyinjiao (SP6) were treated with acupuncture.

Baihui (GV20) is located in the center of the parietal bone in rats, Shuigou (GV26) is located 1 mm below the tip of the rat's cleft lip in the center of the nose. Neiguan (P6) is located on the medial side of the forelimb of the rat, about 3 mm away from the carpal joint between the radial and ulnar suture. Sanyinjiao (SP6) is located about 10 mm straight up from the tip of the inner ankle of the hind limb of the rat. In the acupuncture group, to ensure smooth acupuncture, rats were restrained with a soft cloth, Shuigou point was inserted to a depth of 1‐mm depth, while the other acupoints were inserted vertically at 2 mm, and needles were retained for 20 min. Acupuncture was started from the day after modeling and was administered once a day for 1 week. All mice in the other groups underwent the same handling and restraint.

### 2.4 Assessment of Nerve Injury

Assessment of modified neurological severity scores (mNSSs) was conducted on the rats both before and after treatment. The mNSS ranges from 0 to 18 points, classifying neurological impairment as *severe* (13–18), *moderate* (7–12), *mild* (1–6), and *absent* (0). The evaluators were blinded to the experimental groups.

### 2.5 Infarction Assessment (2,3,5‐Triphenyltetrazolium Chloride [TTC])

Seven days after modeling, all rats underwent brain tissue collection. Six coronal slices, each 1–2 mm thick, were obtained from each isolated brain. For TTC staining, the slices were incubated in TTC staining reagent (2%) at 37°C under dark conditions, followed by fixation in 4% paraformaldehyde. Ischemic (unstained) area quantification and cerebral infarct size calculations were performed using ImageJ (image processing software) by an independent observer blinded to group allocation. The extent of the infarct area in the total brain area was evaluated by the following formula: brain tissue infarct area (%) = cerebral infarct area/total brain area × 100%.

### 2.6 Brain Tissue Extraction for Sequencing

After treatment, the rats received intraperitoneal administration of Zoletil 50 (50 mg/kg) for anesthesia. Normal saline (0.9%) was perfused via the left ventricle until the liver appeared pale. Following brain collection, the brain tissue from the ischemic area was collected, cut into small pieces, and placed in cryopreservation tubes, followed by immediate immersion in liquid nitrogen for sequencing.

### 2.7 RNA Extraction Library Construction and Sequencing

RNA extraction, library preparation, and sequencing were conducted following standardized methods, as described in previous studies (Liu et al. 2024). Trizol reagent (15596018, Thermo Fisher, CA, USA) was applied for total RNA extraction, as instructed by the manufacturer. Using the Bioanalyzer 2100 and RNA 6000 Nano LabChip Kit (5067‐1511, Agilent, CA, USA), the total RNA quantity and purity were evaluated. Sequencing libraries were generated using high‐quality RNA samples with RIN number > 7.0. After extracting total RNA, total RNA (5 µg) was subjected to mRNA purification via Dynabeads Oligo (dT) (Thermo Fisher) through two successive rounds. Then, the Magnesium RNA Fragmentation Module (E6150, NEB, USA) was adopted for mRNA fragmentation into short fragments with divalent cations at 94°C for 5–7 min. Subsequently, using SuperScript II Reverse Transcriptase (1896649, Invitrogen, USA), reverse transcription of cleaved RNA fragments into cDNA was conducted. The resulting cDNA was then utilized for synthesizing U‐labeled second‐stranded DNAs employing dUTP Solution (R0133, Thermo Fisher), RNase H (NEB, cat.m0297, USA) and E. coli DNA polymerase I (m0209, NEB). To prepare for ligation with indexed adapters, an A‐base was incorporated at each strand's blunt ends. A T‐base overhang was present in each adapter to enable its ligation to A‐tailed fragmented DNA. The fragments underwent ligation with dual‐index adapters, after which AMPure XP beads were utilized for size selection. Afterward, the U‐labeled second‐stranded DNAs were exposed to heat‐labile UDG enzyme (m0280, NEB). The ligated products underwent PCR amplification under the following conditions: 95°C for 3 min (initial denaturation); eight cycles of 98°C for 15 s (denaturation), 60°C for 15 s (annealing), and 72°C for 30 s (extension); with a final extension at 72°C for 5 min. The final cDNA libraries exhibited an average insert size of 300 ± 50 bp. Ultimately, by Illumina Novaseq 6000 (LC‐Bio Technology CO., Ltd., Hangzhou, China), the 2 × 150 bp paired‐end sequencing (PE150) was conducted as per the vendor's prescribed procedures.

### 2.8 Data Analysis

Using the “DESeq2” package, bioinformatic analysis of differentially expressed genes (DEGs) across groups was performed. A fold change (FC) ≥ 1.5 and *p* < 0.05 were used as screening thresholds. Through the DAVID (https://david.ncifcrf.gov/) database, enrichment analyses of Gene Ontology (GO) and KEGG pathways were carried out. The STRING database (https://cn.string‐db.org/) was utilized for protein–protein interaction (PPI) analysis.

### 2.9 qRT‐PCR

Using TRIzol reagent (Invitrogen, Carlsbad, CA, United States), brain tissues were processed for total RNA extraction. Following cDNA synthesis with a reverse transcription kit (Takara, Dalian, China), RT‐PCR was conducted via a SYBR Green Master Mix Kit (Takara Bio Inc., China) in a 25 µL reaction system, according to the manufacturer's instructions. The experiment was performed in triplicate. The Cfx96 real‐time system software was utilized for determining each well's CT value, followed by relative FC calculation via the 2^−∆∆CT^ method. Table 1 provides a summary of the primer sequences.

**TABLE 1:** The primers employed in qRT‐PCR.

Target	Forward primer (5′–3′)	Reverse primer (5′–3′)
*C1QA*	CCATATCGCTGGCCTCTATGG	GTCTTCCTGCCTCCCCTTTC
*C1QB*	GGCCACCGACAAGAACTCACTAC	CCATATCTGGAAAGAGCAGGAACC
*C1QC*	AACCAATCAGGTCAACTCGGG	CCCACCATGTCGTAGTAGTCATTG
*C6*	TTCTGGAACCCAGAGCAGA	TGATGGGGCATCTTTGCCAG
*C7*	CAATGAACTCACTGGCCA	CATTTCCACTCAGCCTGT
*C2*	GGGAGTGTCTGAGTAACGGAGTG	CAAGCAGCAGATAGAGGTTCAGG

### 2.10 Western Blot

The Bradford method was utilized for protein concentration quantification, followed by SDS‐PAGE gel preparation, sample loading, electrophoresis, and protein transfer onto a membrane. Following a 1‐h blocking step, the membranes underwent three 10‐min TBST washes before the addition of primary antibodies (against C1qa, C1qc, and C6, 1:1000). The primary antibody was removed the next day after an overnight incubation at 4°C, followed by three rinses in TBST, each lasting 10 min. Then, a 1.5‐h incubation was conducted at ambient temperature with the HRP‐labeled secondary antibody (1:5000). Following thorough washing, the membrane was developed by adding a chromogenic solution and then exposed. The images were saved, and quantitative analysis was conducted using ImageJ software, quantitative analysis was carried out with the actin antibody as the internal control.

### 2.11 Statistical Analysis

SPSS.25 software was used, and mean ± SD was applied for expressing the data. For comparisons between the two groups, the Student's *t*‐test was adopted. A *p*‐value of < 0.05 represented the statistical significance threshold.

## 3 Results

### 3.1 Experimental Flow Chart

**FIGURE 1:**
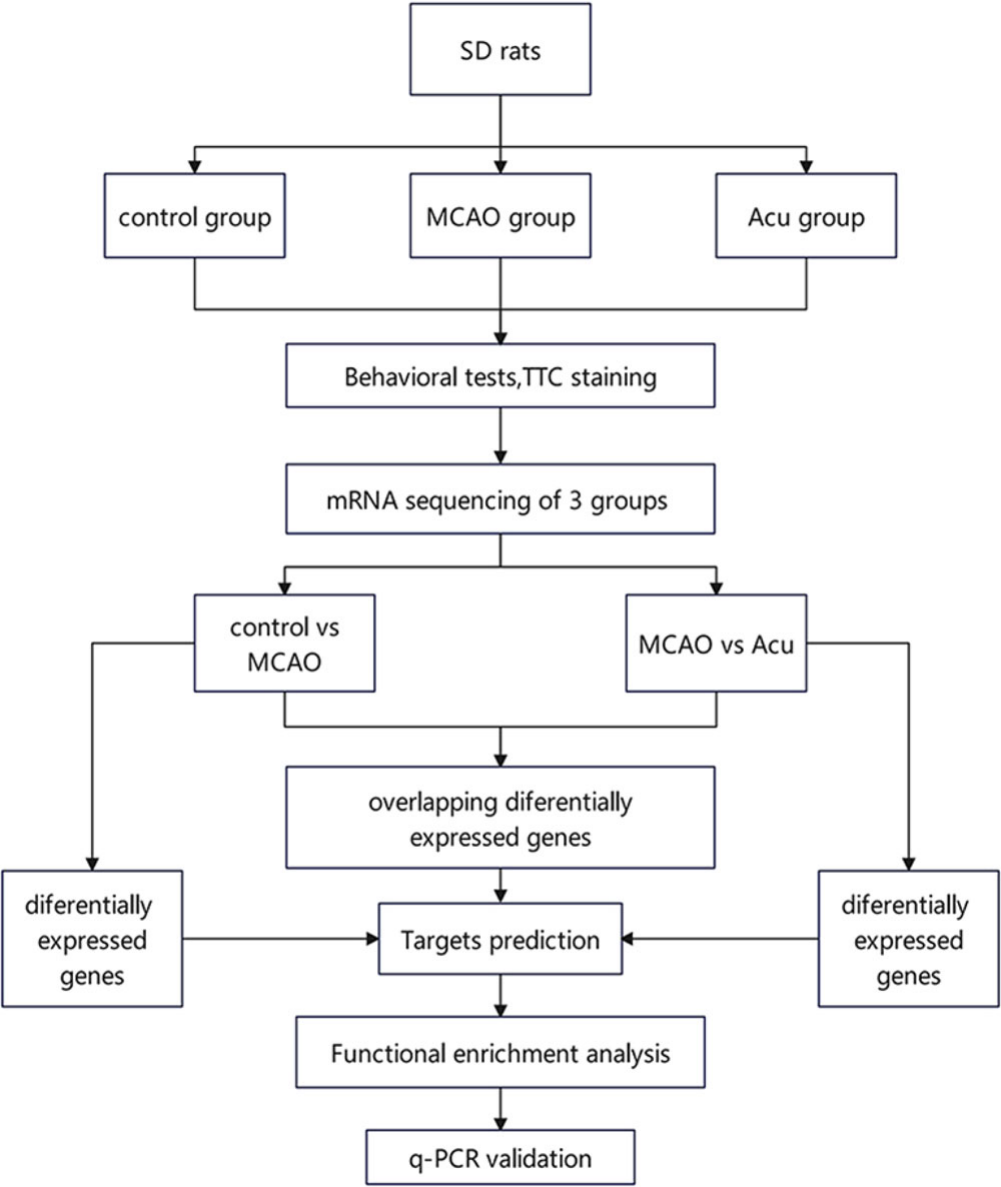
Experimental flow chart.

The experimental flow chart is shown in Figure 1.

### 3.2 Neurological Deficits

**FIGURE 2:**
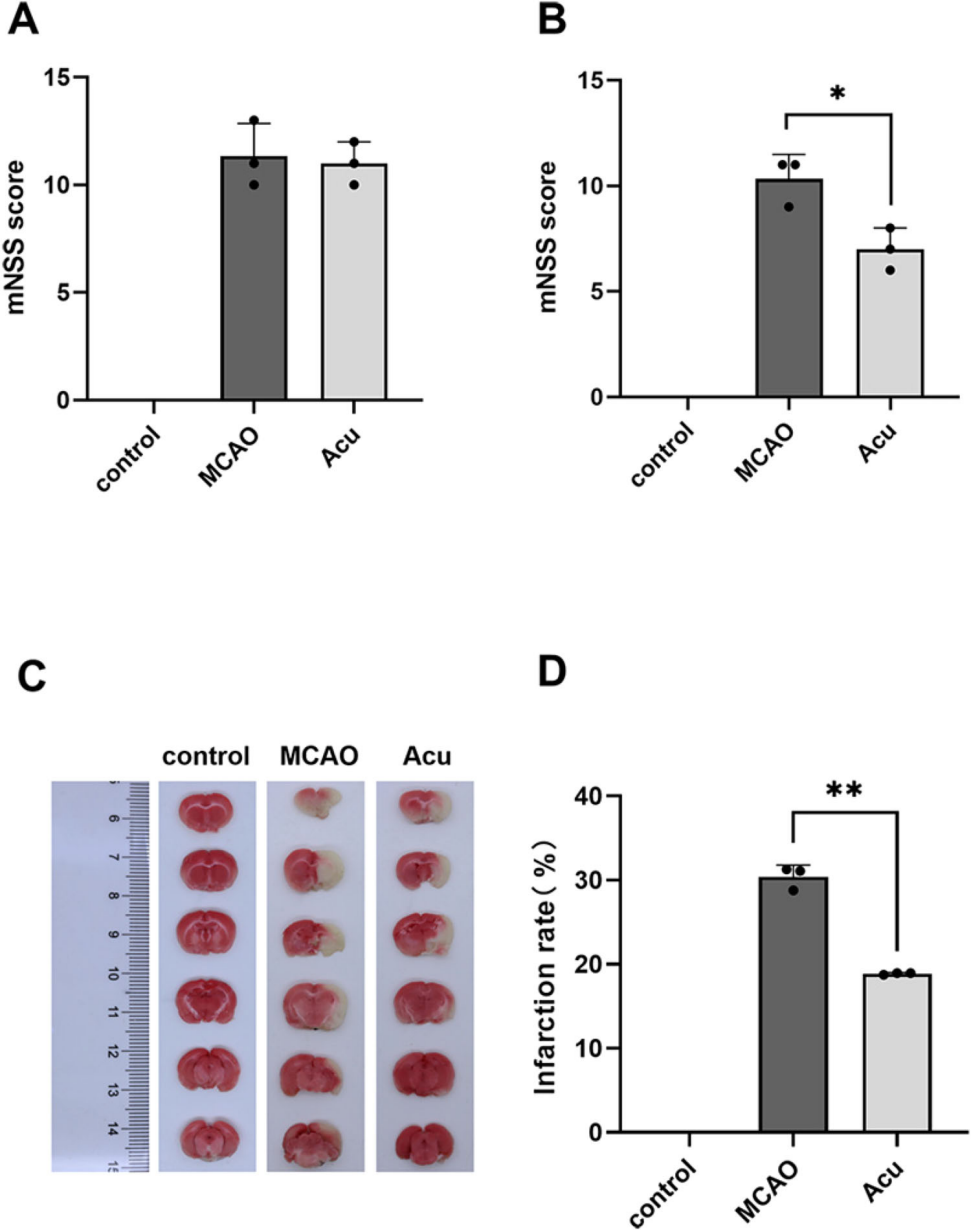
Nerve defect in MCAO rat model. (A) Pretreatment mNSS score. (B) Posttreatment mNSS score. (C) Images of brain sections stained with TTC after treatment. (D) Quantification of infarct size. *N* = 3; the data are shown as mean ± SD. *p < 0.05, **p < 0.01 versus MCAO.

After establishing the MCAO model, the mNSS of each rat was determined for assessing their neurobehavior, including balance, reflex, motor, and sensory tests. The findings revealed that the mNSSs of the MCAO group mice and the acupuncture group mice increased before treatment, with no difference among the groups, suggesting the model's successful establishment and the presence of severe neurological deficits in IS model mice (Figure 2A). After treatment, compared to the MCAO group, the acupuncture group demonstrated a considerably lower score (Figure 2B) (*p* < 0.05), indicating that acupuncture can improve neurological deficits. Moreover, we found that the infarct size was significantly reduced (Figure 2C,D) (*p* < 0.01).

### 3.3 Identification of DEGs

**FIGURE 3:**
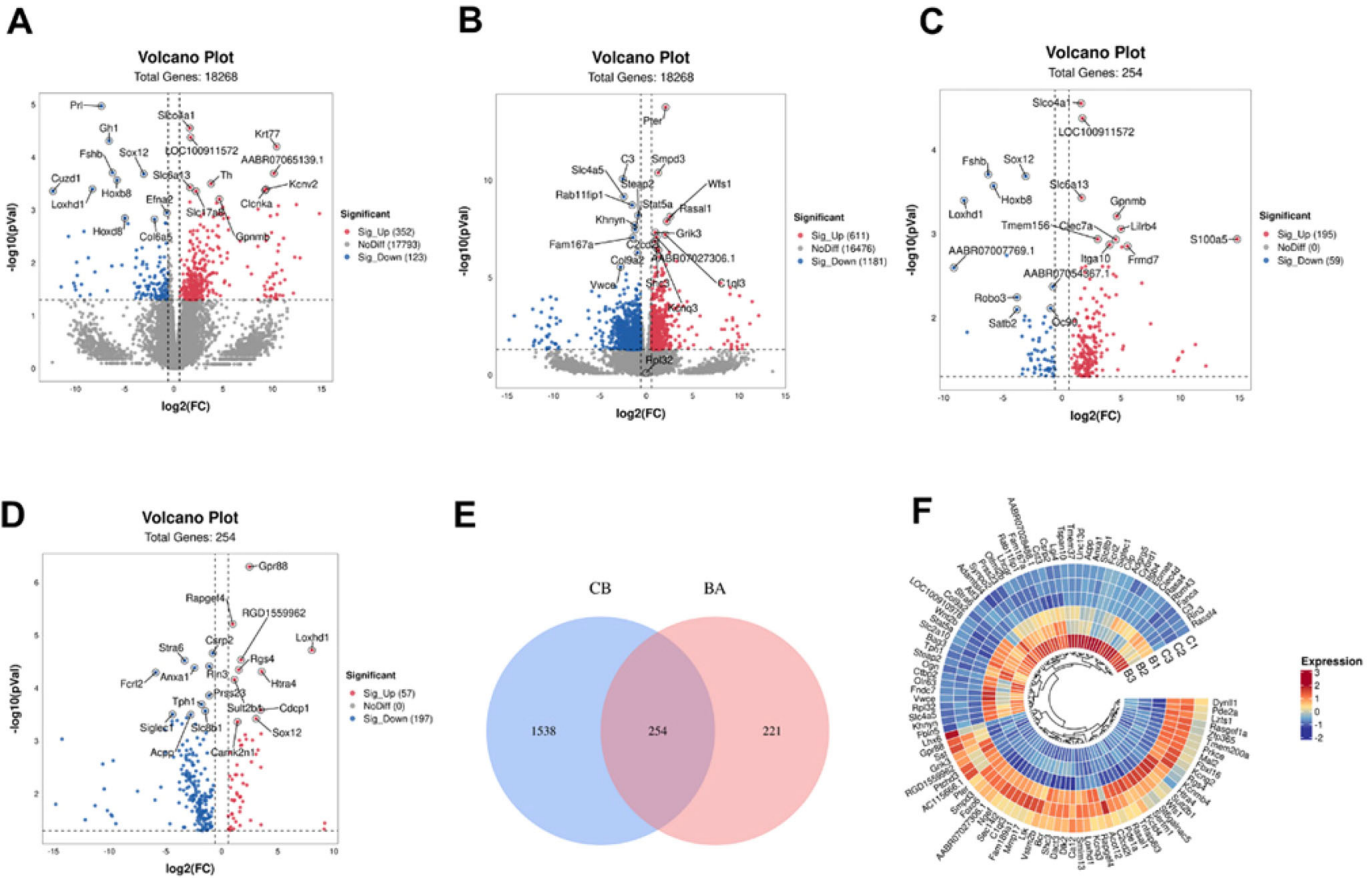
Identification of differentially expressed genes. (A–D) Volcano plots of differentially expressed genes, BA group (A), CB group (B), 254 core genes in BA group (C), 254 core genes in CB group (D). (E) Wayne diagram of DEGs between BA and CB groups. (F) Ring heatmap of the expression of some core genes between CB groups.

RNA‐Seq analysis resulted in the successful identification and mapping of 18,268 genes. DEGs meeting the criteria of *p* < 0.05 and FC ≥ 1.5 were then selected. Between the MCAO and control groups, 475 DEGs were obtained, including 123 downregulated genes and 352 upregulated genes. Moreover, 1792 DEGs were detected between the MCAO group and the acupuncture group, consisting of 1181 downregulated genes and 611 upregulated genes (Figure 3A,B). The 10 most significantly upregulated and 10 most significantly downregulated genes were marked.

We compared the DEGs and identified 254 hub genes that best reflected the effect of acupuncture (Figure 3E). Volcano plots in Figure 3C,D show the distributions of these hub genes among the three groups. Between the control and MCAO groups, 59 downregulated genes were identified, accompanied by 195 upregulated genes. Additionally, the MCAO and acupuncture groups exhibited 197 downregulated genes as well as 57 upregulated genes. Similarly, the most significantly upregulated and most significantly downregulated genes were marked. Moreover, Figure 3F displays these hub gene expression levels between the MCAO and acupuncture groups.

### 3.4 KEGG and GO Enrichment Analyses of the DEGs

**FIGURE 4:**
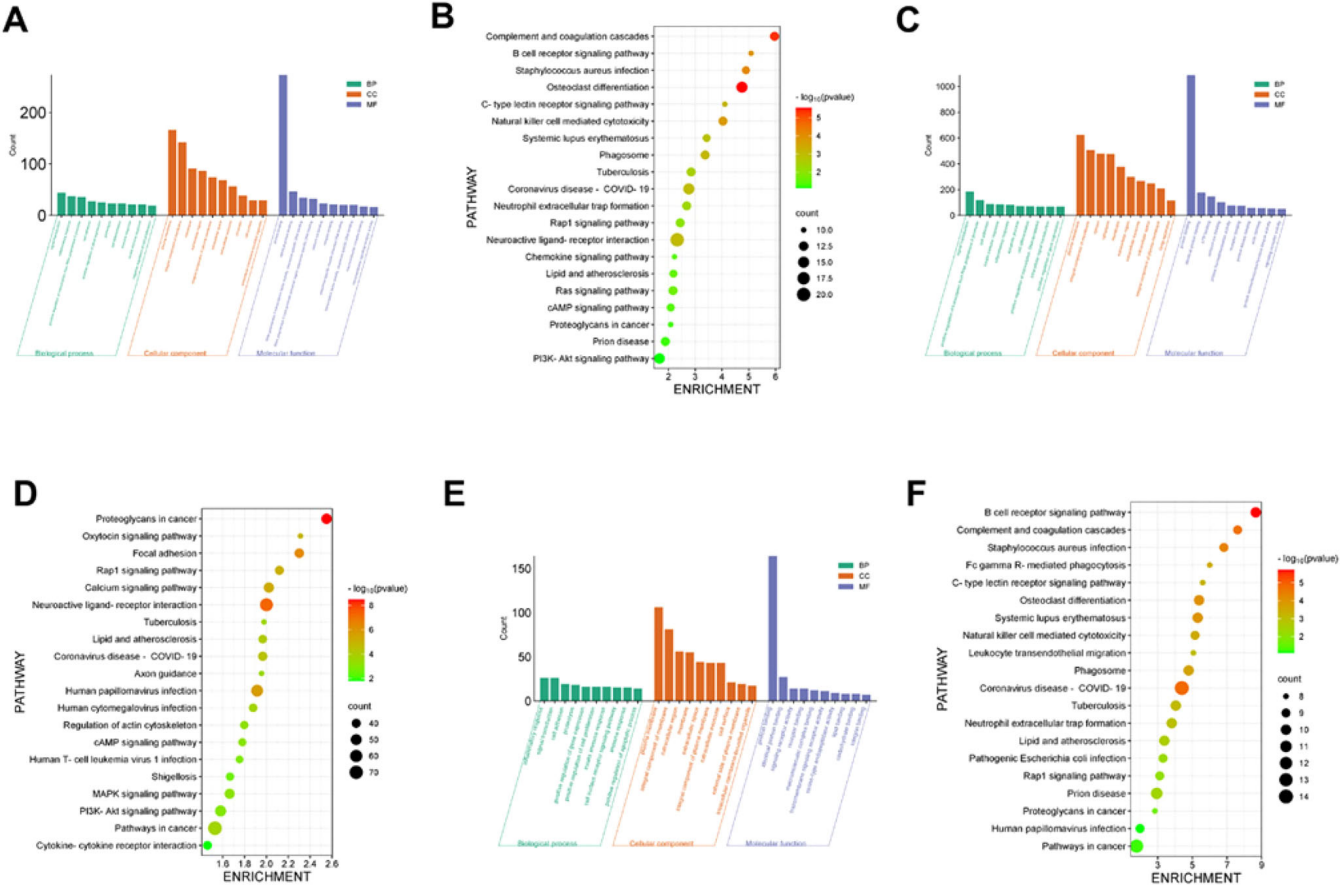
GO and KEGG analysis of differentially expressed genes. GO (A) and KEGG (B) enrichment analyses of DEGs between BA groups. GO (C) and KEGG (D) enrichment analyses of DEGs between CB groups. GO (E) and KEGG (F) enrichment analyses of 254 core genes.

GO and KEGG enrichment analyses were applied to the DEGs across groups to further analyze the molecular mechanisms involved in stroke treatment by acupuncture. GO analysis encompassed biological process (BP), molecular function (MF), and cellular composition (CC), with the top 10 enriched terms in the DEGs (*p* < 0.05) displayed (Figure 4A,C). KEGG analysis revealed that 41 pathways were enriched in the DEGs among groups (Figure 4B,D). The top 20 enriched in the DEGs with *p* < 0.05 were displayed. The 254 hub genes underwent GO and KEGG enrichment analyses (Figure 4E,F). These results may better represent the molecular mechanisms and pathways related to the effect of acupuncture.

Through GO analysis of the mechanisms of stroke and acupuncture treatment, we found that the enriched BPs in the DEGs between the acupuncture and stroke groups were all highly related to inflammation, such as signal transduction, inflammatory response, cell adhesion, immune response, and innate immune response, and the GO enrichment analysis of the 254 hub genes showed similar results.

According to KEGG enrichment analysis, the signaling pathways associated with stroke pathogenesis and those related to the effect of acupuncture on stroke (254 hub genes) were generally consistent, and the enriched pathways included the complement and coagulation cascades, C‐type lectin receptor signaling pathways, B‐cell receptor signaling pathways, Ras signaling pathways, PI3K‐Akt signaling pathways, chemokine signaling pathways, cAMP signaling pathways, and those involved in natural killer cell‐mediated cytotoxicity, the formation of neutrophil extracellular traps (NETs), systemic lupus erythematosus, phagocytes, coronavirus disease —COVID‐19, neutrophils, and *Staphylococcus aureus* infection.

### 3.5 PPI Analysis

**FIGURE 5:**
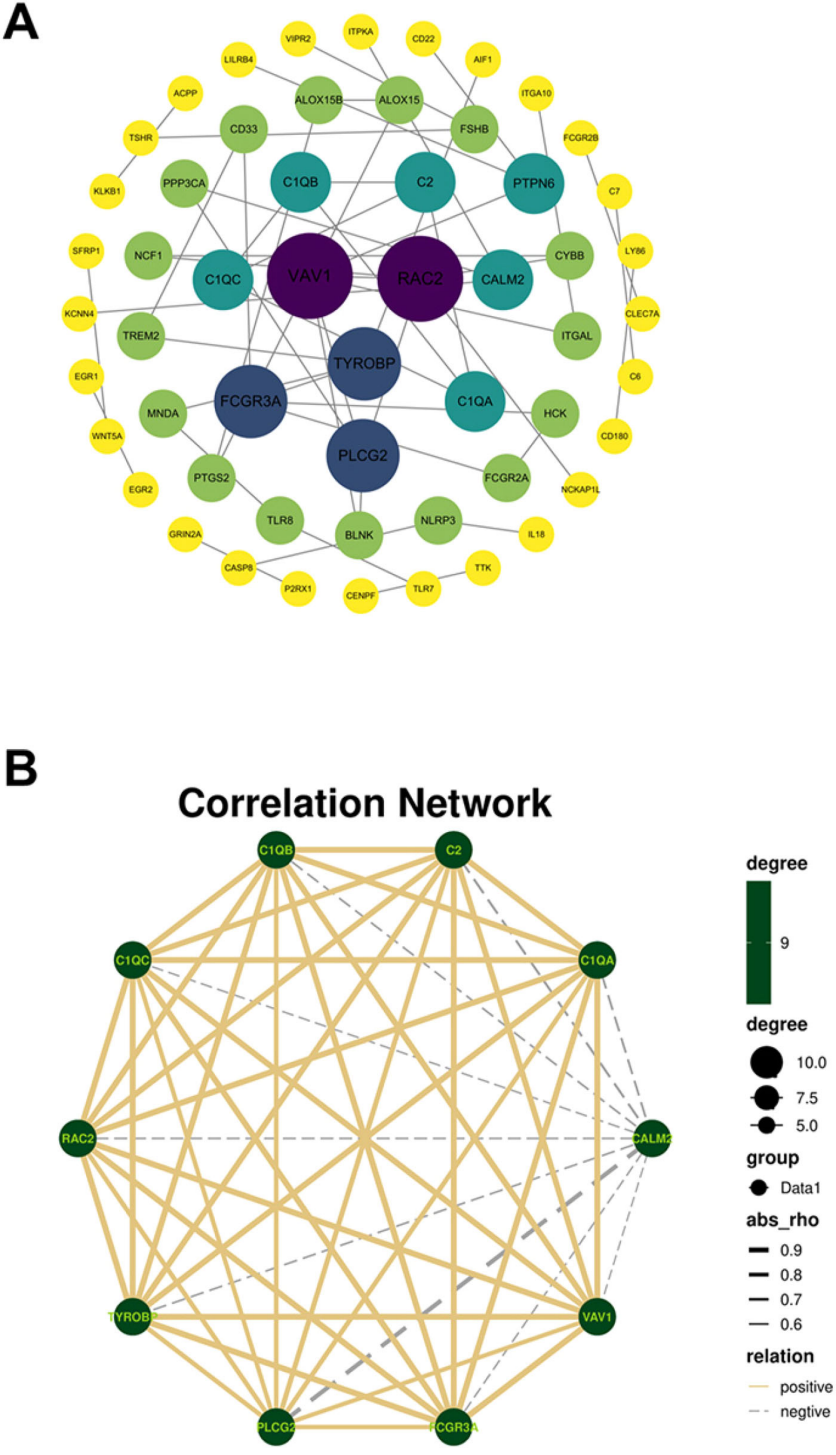
PPI analysis. (A) Protein–protein interaction network. (B) Correlation analysis of 10 hub genes.

We imported 254 hub genes into the STRING database and selected “highest confidence (0.9)” as the screening threshold. Additionally, Cytoscape software was utilized for constructing a PPI network (Figure 5A). The PPI network comprised 50 edges and 55 nodes. We screened 10 hub genes, namely, VAV1, RAC2, TYROBP, PLCG2, FCGR3A, C1QA, C2, C1QB, C1QC, and CALM2. Based on the Hub gene expression, a correlation analysis was conducted among the groups (Figure 5B). The expression of genes other than CALM2 was positively correlated with each other, whereas CALM2 displayed a negative correlation with the other nine genes.

### 3.6 Expression of Complement‐Related Genes in the Blank, MCAO, and Acupuncture Groups

**FIGURE 6:**
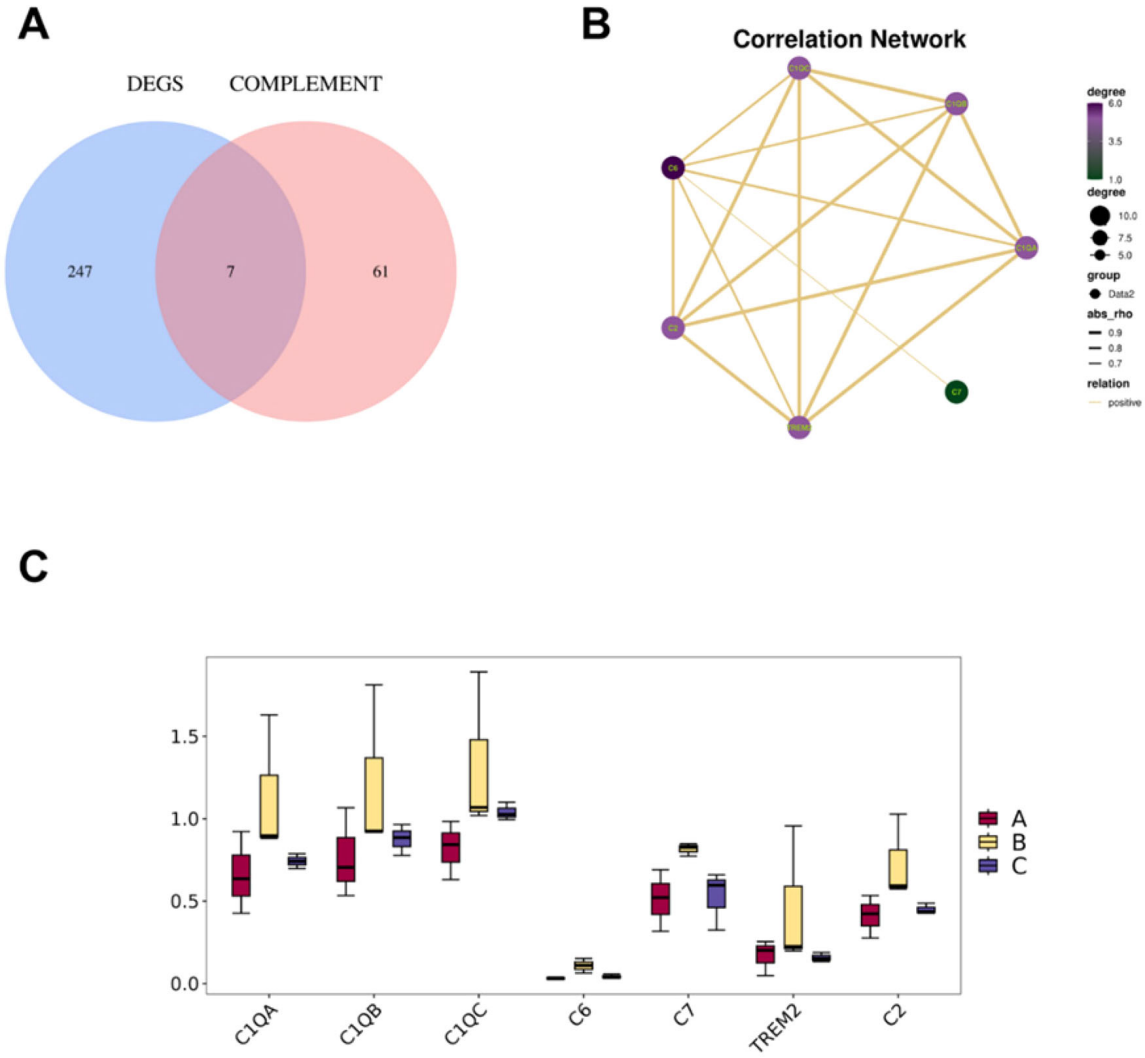
Expression of complement‐related genes in groups A, B, and C. (A) Wayne plots of the 254 core genes with complement‐related genes. (B) Correlation analysis of seven complement genes associated with acupuncture treatment. (C) Box and line plots showing their expression in groups A, B, and C.

Through gene set enrichment analysis (GSEA), we obtained 68 complement‐related genes and compared them with the 254 hub genes (Figure 6A). The common genes were identified as complement genes related to acupuncture treatment and included C1QA, C1QB, C1QC, C6, C7, TREM2, and C2. Box plots depict these seven complement gene expression levels in each group C. As shown in Figure 6C, these seven complement‐related genes were overexpressed during cerebral ischemia‒reperfusion, and acupuncture significantly downregulated their expression. Correlation analysis revealed a positive correlation among these seven complement‐related gene expression levels (Figure 6B).

**FIGURE 7:**
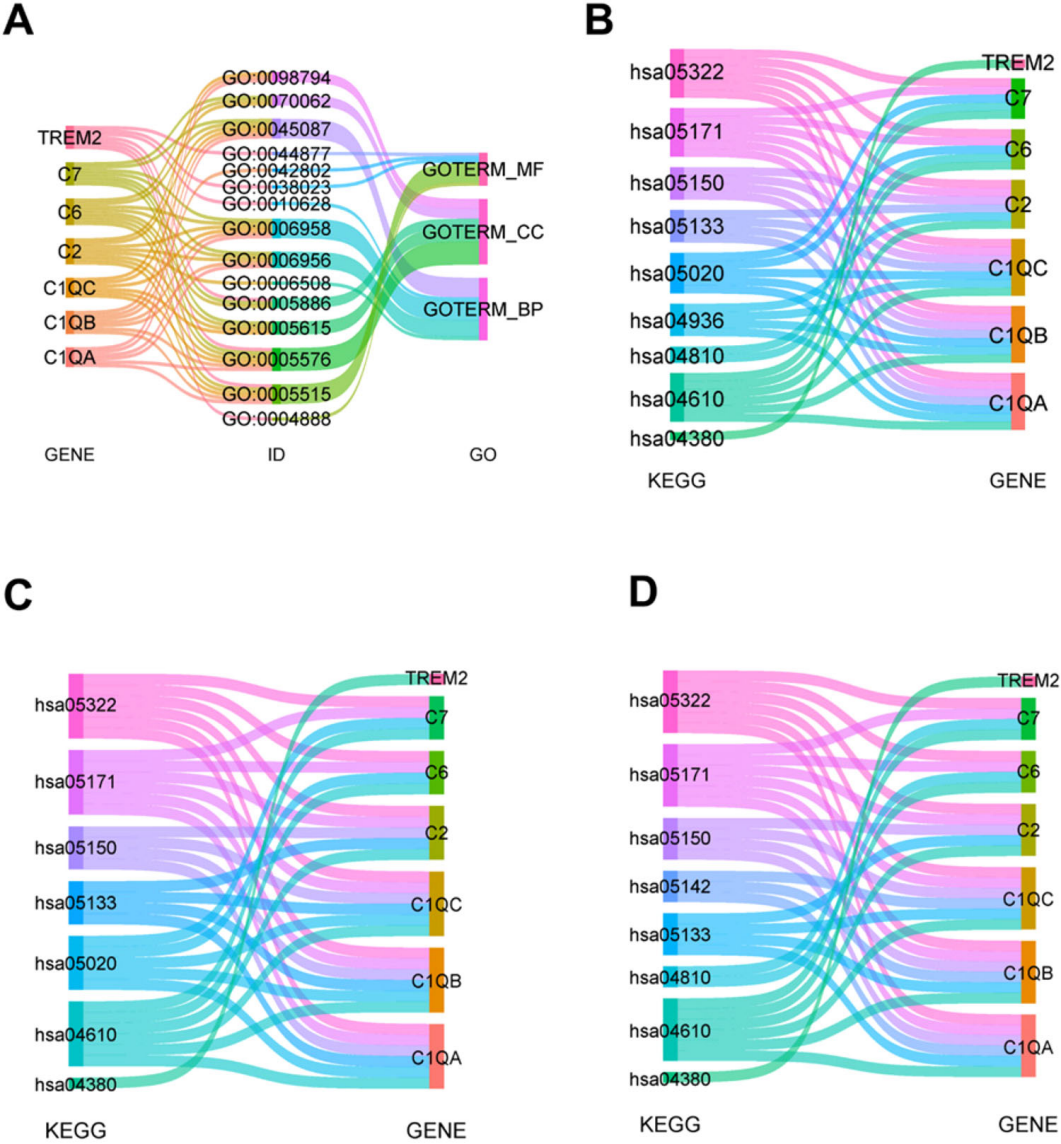
Expression of complement‐related genes in groups A, B, and C. (A–B) Labeling of seven complement genes in the GO (A) and KEGG (B) enrichment analysis results of 254 core genes. (C) Labeling of seven complement genes in the KEGG enrichment analysis results of DEGs between BA groups (C), as well as between CB groups (D).

Next, we compared these seven complement‐related genes to the GO and KEGG entries enriched in the 254 hub genes, and the GO and KEGG entries involved were listed in Tables 2 and 3. The Sankey diagram (Figure 7A,B) shows the connection between the seven complement‐related genes and the GO and KEGG entries in detail. We also compared the seven complement‐related genes to the KEGG pathways enriched in the DEGs among the groups. The detailed information is listed in Tables 4 and 5 and in the Sankey diagram (Figure 7C,D).

**TABLE 2:** Tagging of seven complement genes in the results of KEGG of 254 core genes.

Pathway ID	Name	Genes
hsa05171	Coronavirus disease—COVID‐19	*C1QB*, *C1QA*, *C2,C6*, *C7*, *CIQC*
hsa05020	Prion disease	*C1QB*, *C1QA*, *C6*,*C7*, *C1QC*
hsa04380	Osteoclast differentiation	*TREM2*
hsa05322	Systemic lupus erythematosus	*C1QB*, *C1QA*, *C6*, *C7*, *C1QC*, *C2*
hsa04610	Complement and coagulation cascades	*C1QB*, *C1QA*, *C6*, *C7*, *C1QC*, *C2*
hsa05150	*Staphylococcus aureus* infection	*C1QB*, *C1QA*, *C1QC*, *C2*
hsa04810	Regulation of actin cytoskeleton	*C6*, *C7*
hsa05133	Pertussis	*C1QB*, *C1QA*, *C1QC*, *C2*
hsa04936	Alcoholic liver disease	*C1QB*, *C1QA*, *C2*, *C1QC*

**TABLE 3:** Seven complement genes were identified in KEGG enrichment analysis of DEGs between BA groups.

Pathway ID	Name	Genes
hsa04380	Osteoclast differentiation	*TREM2*
hsa05171	Coronavirus disease—COVID‐19	*C1QA*, *C1QB*, *C1QC*, *C6*, *C7*, *C2*
hsa04610	Complement and coagulation cascades	*C1QB*, *C1QA*, *C6*, *C7*, *C1QC*, *C2*
hsa05020	Prion disease	*C1QB*, *C1QA*, *C6*, *C7*, *C1QC*
hsa05150	*Staphylococcus aureus* infection	*C1QB*, *C1QA*, *C1QC*, *C2*
hsa05322	Systemic lupus erythematosus	*C1QB*, *C1QA*, *C6,C7*, *C1QC*, *C2*
hsa05133	Pertussis	*C1QB*, *C1QA*, *C1QC*, *C2*

**TABLE 4:** Labeling of seven complement genes in the results of KEGG enrichment analysis of DEGs between CB groups.

Pathway ID	Name	Genes
hsa05171	Coronavirus disease—COVID‐19	*C1QA*, *C1QB*, *C1QC*, *C6*, *C7*, *C2*
hsa04810	Regulation of actin cytoskeleton	*C6*, *C7*
hsa04380	Osteoclast differentiation	*TREM2*
hsa05133	Pertussis	*C1QB*, *C1QA*, *C2*, *C1QC*
hsa04610	Complement and coagulation cascades	*C1QB*, *C1QA*, *C2*, *C6*, *C7*, *C1QC*
hsa05142	Chagas disease	*C1QB*, *C1QA*, *C1QC*
hsa05322	Systemic lupus erythematosus	*C1QB*, *C1QA*, *C2*, *C6*, *C7*, *C1QC*
hsa05150	*Staphylococcus aureus* infection	*C1QB*, *C1QA*, *C2*, *C1QC*

**TABLE 5:** Labeling of seven complement genes in the results of KEGG enrichment analysis of DEGs between CB groups.

Pathway ID	Name	Genes
hsa05171	Coronavirus disease—COVID‐19	*C1QA*, *C1QB*, *C1QC*, *C6*, *C7*, *C2*
hsa04810	Regulation of actin cytoskeleton	*C6*, *C7*
hsa04380	Osteoclast differentiation	*TREM2*
hsa05133	Pertussis	*C1QB*, *C1QA*, *C2*, *C1QC*
hsa04610	Complement and coagulation cascades	*C1QB*, *C1QA*, *C2*, *C6*, *C7*, *C1QC*
hsa05142	Chagas disease	*C1QB*, *C1QA*, *C1QC*
hsa05322	Systemic lupus erythematosus	*C1QB*, *C1QA*, *C2*, *C6*, *C7*, *C1QC*
hsa05150	*Staphylococcus aureus* infection	*C1QB*, *C1QA*, *C2*, *C1QC*

According to the GO analysis results of the 254 hub genes, acupuncture affected IS mainly through the regulation of BPs including the classical pathway of complement activation, complement‐involved proteolysis, complement activation, positive regulation of gene expression, and the innate immune response. Given the KEGG analysis of complement among the 254 hub genes, acupuncture ameliorated IS mainly through the regulation of pathways such as those involved in complement‐mediated osteoclast differentiation, the complement coagulation cascade, *Staphylococcus aureus* infection, and systemic lupus erythematosus.

### 3.7 qPCR and Western Blot

**FIGURE 8:**
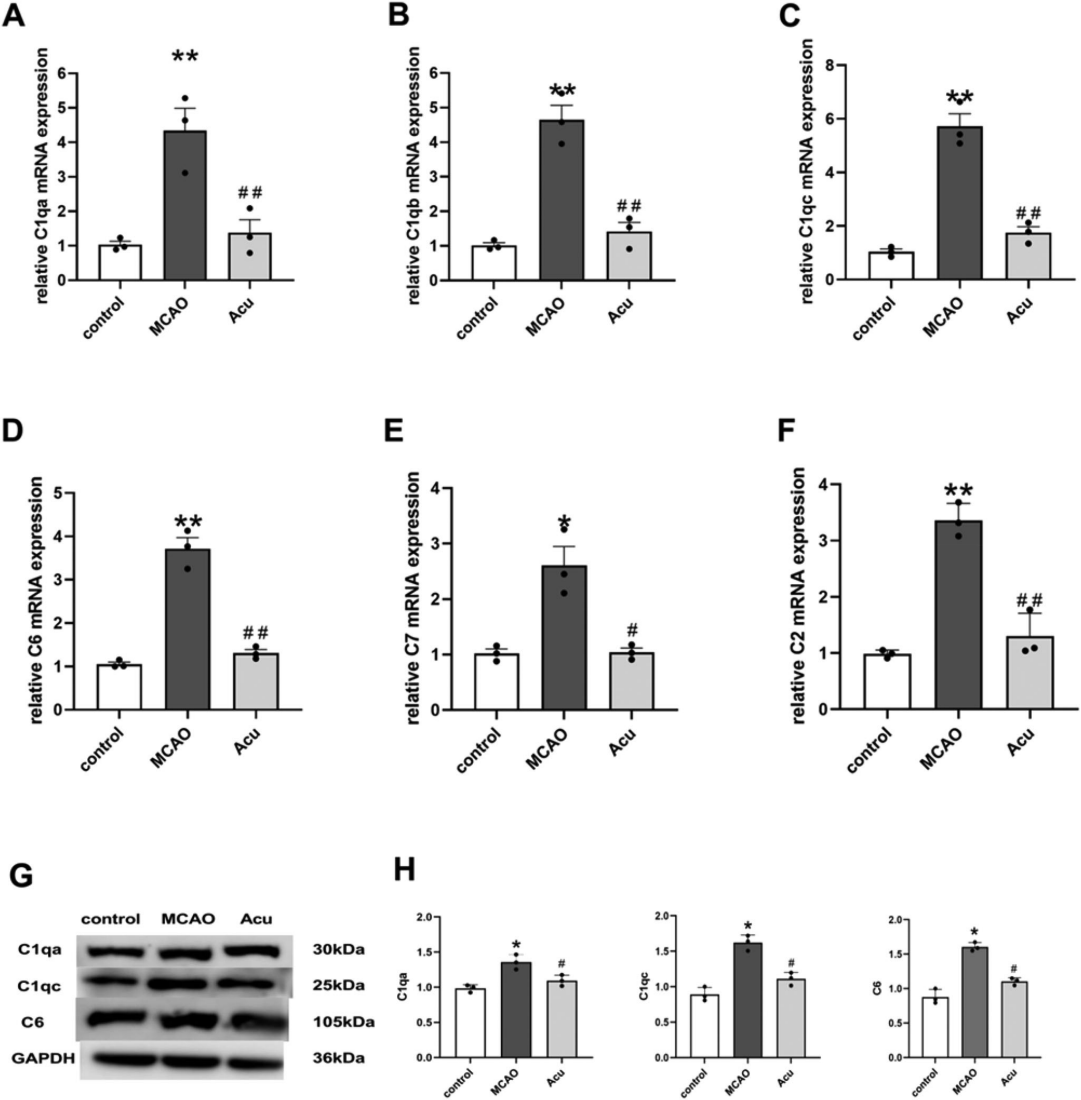
Validation of complement‐related gene and protein expression. (A–F) qRT‐PCR verification of complement‐related gene (C1qa, C1qb, C1qc, C6, C7, and C2) mRNA expression across the control, MCAO, and acupuncture (acu) groups. Data are shown as mean ± SD (*n* = 3). (G–H) Western blot analysis of C1qa, C1qc, and C6 protein expression in the three groups. *p < 0.05, **p < 0.01 versus control; #*p* < 0.05, ##*p* < 0.01 versus MCAO.

We validated the six complement‐related genes (C1qa, C1qb, C1qc, C6, C7, and C2) by qPCR. The qPCR results revealed marked upregulation of all six complement‐related genes in the MCAO group, whereas their expression was significantly reduced in the acupuncture group. We validated the three complement‐related proteins (C1qa, C1qc, and C6) by Western blot. The results showed that all protein expression trends coincide with qPCR (Figure 8). Hence, the reliability of the RNA sequencing results was validated by qPCR analysis.

## 4 Discussion

This study systematically investigated the therapeutic mechanisms of acupuncture in alleviating ischemic brain injury using a rat model of MCAO. Transcriptomic analysis revealed 1792 DEGs between the MCAO and acupuncture‐treated groups, with 254 identified as hub genes reflecting acupuncture responsiveness. GO and KEGG analyses highlighted the complement cascade as a central pathway modulated by acupuncture. Specifically, acupuncture significantly downregulated the expression of key complement‐related genes, including C1QA, C1QB, C1QC, C2, C6, and C7. These findings suggest that acupuncture mitigates ischemia‐induced neuroinflammation and tissue injury primarily through modulation of the complement system and associated inflammatory responses.

The complement system is a central component of innate immunity and is strongly activated in cerebral ischemia–reperfusion injury. Enrichment analysis revealed that several DEGs were significantly involved in the “complement and coagulation cascade” pathway (KEGG hsa04610). The classical pathway, initiated by C1q‐a protein composed of C1QA, C1QB, and C1QC subunits, is a major contributor to stroke‐induced inflammation and vascular injury. C1q activates microglia and astrocytes, enhances pro‐inflammatory signaling, and disrupts blood–brain barrier integrity (Elvington et al. 2012; Heydenreich et al. 2012; Ten et al. 2010). Furthermore, C2 participates in C3 convertase formation, amplifying complement activation, while C6 and C7 are key components of the membrane attack complex (MAC), which promotes cell lysis and inflammation (Laudisi et al. 2013; Aarsetøy et al. 2021). The observed downregulation of these genes following acupuncture implies a direct role of acupuncture in inhibiting complement overactivation, thereby alleviating neurovascular damage and inflammatory responses.

Beyond the complement cascade, acupuncture influenced several other critical signaling pathways, including the PI3K‐Akt pathway, NET formation, and calcium signaling. The PI3K‐Akt pathway is known to promote neuronal survival and repair following ischemic injury (Denorme et al. 2022). NETs, released by infiltrating neutrophils, have been implicated in thrombosis and microvascular occlusion during stroke (Denorme et al. 2022; Kang et al. 2020). Acupuncture may inhibit NET formation, thus improving reperfusion and reducing infarct size. Calcium signaling, involved in endothelial permeability and immune cell activation, also appears to be modulated. Together, these findings indicate that acupuncture exerts a broad‐spectrum regulatory effect by targeting multiple pathways that converge on inflammation, thrombosis, and tissue remodeling.

To confirm transcriptomic findings, the expression of six complement‐related genes (C1qa, C1qb, C1qc, C2, C6, and C7) was validated by qRT‐PCR, and three proteins (C1qa, C1qc, and C6) were confirmed by western blot analysis. The expression trends were consistent with RNA‐seq data, demonstrating increased expression in the MCAO group and significant suppression following acupuncture treatment. These results align with existing literature, where inhibition of C1q has been shown to reduce infarct size and blood–brain barrier permeability (Heydenreich et al. 2012; Schartz and Tenner 2020), and deficiencies in C6 and C7 mitigate inflammatory and thrombotic responses (Laudisi et al. 2013; Aarsetøy et al. 2021; Han et al. 2019). The downregulation of C2, previously associated with endothelial dysfunction during ischemic conditions, further underscores acupuncture's anti‐inflammatory and vascular protective effects (Wang et al. 2020).

This study provides novel insights into the molecular mechanisms of acupuncture in ischemic stroke, particularly its association with complement system modulation. While the observed downregulation of complement components is encouraging, further functional studies are needed to clarify the precise regulatory relationship. Additionally, the origin of these components—whether centrally produced or peripherally derived—and the temporal effects of acupuncture warrant deeper investigation, potentially through cell‐specific and time‐course approaches. Although the MCAO model is widely used and well‐validated, certain differences from human stroke should be considered when interpreting translational relevance. Overall, these findings highlight the complement system as a promising target for acupuncture‐based interventions, meriting continued exploration.

## 5 Conclusion

Our study suggests that acupuncture may influence the complement system, as evidenced by transcriptomic and protein expression changes, and this modulation is associated with reduced inflammatory responses in ischemic stroke. However, further experiments, such as those using complement inhibitors, are necessary to confirm a causal relationship. In addition, acupuncture may affect stroke outcomes through mechanisms potentially involving the complement‐related coagulation response, blood–brain barrier permeability, NET formation, and calcium signaling pathways, although these mechanisms require further experimental validation. Our results revealed that acupuncture, as an adjuvant therapy for stroke, may exert its therapeutic effect by reducing inflammation through the regulation of the complement system.

## Author Contributions

**Yanlin Liu**: conceptualization, investigation, funding acquisition, writing – original draft, writing – review and editing, visualization, validation, methodology, project administration, formal analysis, software, resources, supervision. **Yuting Jin**: investigation, visualization, formal analysis, supervision, writing – review and editing. **Yuxin Liu**: investigation, visualization, formal analysis, supervision, writing – review and editing. **Jiaqi Gao**: investigation, validation, formal analysis, supervision, writing – review and editing. **Xiaomei Wang**: investigation, visualization, project administration, writing – review and editing. **Hongbin Ren**: investigation, methodology, software, writing – review and editing. **Yabo Hao**: investigation, validation, project administration, writing – review and editing. **Xibin Yang**: conceptualization, investigation, funding acquisition, writing – original draft, writing – review and editing, visualization, validation, methodology, project administration, formal analysis, software, resources, supervision, data curation. **Kaitao Luo**: data curation, supervision, resources, project administration, formal analysis, software, methodology, validation, visualization, writing – review and editing, writing – original draft, funding acquisition, investigation, conceptualization.

## Ethics Statement

This study was approved by the Laboratory Animal Management and Welfare Ethical Review Committee of Zhejiang Yingyang Pharmaceutical Research Co., Ltd., with the approval number ZJEY‐20230529‐07. All procedures were performed in accordance with the Guide for the Care and Use of Laboratory Animals of the National Institutes of Health.

## Consent

The authors have nothing to report.

## Conflicts of Interest

The authors declare no conflicts of interest.

## Peer Review

The peer review history for this article is available at https://publons.com/publon/10.1002/brb3.70740.

## Data Availability

The data that support the findings of this study are available from the corresponding author upon reasonable request.
